# A decision support system for upper limb rehabilitation robot based on hybrid reasoning with RBR and CBR

**DOI:** 10.3389/fbioe.2024.1400912

**Published:** 2024-04-24

**Authors:** Sujiao Li, Shuhan Xiang, Qiqi Ma, Wenqian Cai, Suiyi Liu, Fanfu Fang, Hongliu Yu

**Affiliations:** ^1^ Institute of Rehabilitation Engineering and Technology, University of Shanghai for Science and Technology, Shanghai, China; ^2^ Shanghai Engineering Research Center of Assistive Devices, Shanghai, China; ^3^ Department of Medical Engineering, Shanghai Eastern Hepatobiliary Surgery Hospital, Naval Medical University, Shanghai, China; ^4^ Changhai Hospital, Shanghai, China

**Keywords:** upper limb rehabilitation robot, decision support system, hybrid reasoning, case-based reasoning, rule-based reasoning

## Abstract

The rehabilitation robot can assist hemiplegic patients to complete the training program effectively, but it only focuses on helping the patient’s training process and requires the rehabilitation therapists to manually adjust the training parameters according to the patient’s condition. Therefore, there is an urgent need for intelligent training prescription research of rehabilitation robots to promote the clinical applications. This study proposed a decision support system for the training of upper limb rehabilitation robot based on hybrid reasoning with rule-based reasoning (RBR) and case-based reasoning (CBR). The expert knowledge base of this system is established base on 10 professional rehabilitation therapists from three different rehabilitation departments in Shanghai who are enriched with experiences in using desktop-based upper limb rehabilitation robot. The rule-based reasoning is chosen to construct the cycle plan inference model, which develops a 21-day training plan for the patients. The case base consists of historical case data from 54 stroke patients who underwent rehabilitation training with a desktop-based upper limb rehabilitation robot. The case-based reasoning, combined with a Random Forest optimized algorithm, was constructed to adjust the training parameters for the patients in real-time. The system recommended a rehabilitation training program with an average accuracy of 91.5%, an average AUC value of 0.924, an average recall rate of 88.7%, and an average F1 score of 90.1%. The application of this system in rehabilitation robot would be useful for therapists.

## 1 Introduction

Stroke is a type of disease in which the blood circulation in the brain is impaired due to cerebrovascular disease, resulting in pathological changes such as ischemia, hypoxia, necrosis, or hemorrhage of brain tissue (Chao et al., 2021). Stroke is often accompanied by limb motor dysfunction occurs, statistics show that stroke patients have a 55%–75% chance of suffering from motor dysfunction (Chen et al., 2022), of which 80% of patients with upper limb dysfunction (Dhiman et al., 2018). The lack of upper limb function makes it difficult for patients to perform activities of daily living independently, which leads to serious family and social issues (Jiang et al., 2023), thus it is necessary to cooperate with rehabilitation training to treat or reduce the upper limb motor dysfunction caused by strokes (Dunkelberger et al., 2020). An effective decision-making prescription for rehabilitation training plays an important role in improving the motor function of stroke patients (LI et al., 2020). The current upper limb rehabilitation robot, which has been able to effectively complete the training program for hemiplegic patients according to the therapist’s rehabilitation decisions. but only focuses on assisting the patient’s training process (Dunkelberger et al., 2020). And the development of rehabilitation training prescription still relies on the clinical experience and scale evaluation results of therapists, which is subjective and has a low degree of standardization. Therefore, decision support system (DSS) has been introduced into stroke rehabilitation training to improve the efficiency and accuracy of rehabilitation training programs.

Currently, the construction methods of decision-making model include rule-based reasoning (RBR), case-based reasoning (CBR) and machine learning generally. RBR is a rule base that summarizes the knowledge of experts in a certain domain, including problem descriptions and solutions, which simulates the reasoning and thinking process of experts in solving professional problems (Huang et al., 2020). Rule base is commonly used as the main knowledge base construction method in the study of intelligent decision-making systems in early upper limb rehabilitation training. Pradeep Natarajan (Natarajan et al., 2011) employed RBR to craft an expert system using CLIPS (C Language Integrated Production System), conducting a survey involving over 100 clinicians and establishing a knowledge repository for a specialized robotic rehabilitation system. Their developed system assists therapists in analyzing data gathered by the rehabilitation robot during training, facilitating decision-making concerning the patient’s rehabilitation process. Douglas D. Dankel (Dankel and Kristmundsdóttir, 2005) developed a post-stroke rehabilitation expert system known as REPS, which utilized the RBR method to create training plans based on assessment scales. Yuan Wang (Yuan, 2015) established an expert system for upper limb rehabilitation robot based on RBR. The system constructed a knowledge base using symptoms and Brunnstrom scales, aiding therapists in decision-making by assessing the patient’s current stage of rehabilitation. Kaixuan Lu (Lu, 2021) established a rule base that amalgamates five evaluation metrics, including muscle tone. This assists in patient rehabilitation by generating output regarding the angle and speed of movement for the rehabilitation robot. The advantage of RBR is that it can summarize a more scientific rehabilitation training plan by referring to the treatment experiences and ideas of several physicians, but it is overly dependent on the established rules, which makes it difficult to formulate the most appropriate training plan for different types of patients.

CBR involves searching historical cases for similarities based on the target case information, wherein these resemblant cases offer solutions to the problem posed by the target case (Slam et al., 2020; Khan and Khan, 2021). At present, more and more studies on the decision-making mechanism of training prescription are based on the case data collected from hospitals, and the decision-making is made through the way of CBR. Meng Lingwei (Lingwei, 2016) searched the patient case information base using a similar patient discovery algorithm based on a conceptual classification tree, considered the patient’s past medical history, family history, and medication use, obtained the weights of each evaluation criterion, and ranked the rehabilitation programs. Chen Ming (Ming et al., 2021) proposed a rehabilitation program recommendation system based on a hybrid attention mechanism neural network model in 2021, which employs an attention mechanism to express the semantic relationship between case text content, and case text. The CBR approach can obtain training programs for new patients from previous cases, which greatly utilizes the medical resources, and does not adhere to the rules and regulations, which makes the development of training programs more flexibility. However, this approach is sensitive to noisy data, and the error and redundant data will have a greater impact on the retrieval efficiency and results, thus CBR method is appropriate for domains characterized by ambiguous knowledge that is challenging to represent through rules.

Therefore, more and more researches tend to combine the two organically and establish a hybrid reasoning mechanism with CBR as the main and RBR as the supplement. In the study of Ji Wen (Wen et al., 2014). They designed a DSS that fused RBR and CBR to control the robot to rehabilitate patients with different speeds and positions, resulting in a functional value of 94 points or more (on a 100-point scale) after rehabilitation treatment. However, their system inadequately utilized and extracted data, posing a challenge in handling large-scale data——a common flaw in training systems integrating RBR and CBR.

Thus, in this study, through the fusion of RBR and CBR, we integrated a machine learning approach into CBR, extensively extracting valuable insights from clinical case data utilizing the random forest algorithm. Ultimately, we created an intelligent decision-making system that constructs a dual-driven hybrid reasoning model, integrating rule-based reasoning and machine learning-enhanced case-based reasoning. This system can adaptively adjust the rehabilitation program based on the patient’s real-time rehabilitation process, enabling the customization of personalized, real-time, and dynamic training programs across multiple rehabilitation stages.

In this study, based on the fusion of the RBR method and the CBR method, we innovatively combined CBR and machine learning algorithms to design a dual-driven hybrid inference model based on RBR and machine learning-enhanced CBR, which achieves the customization of personalized, real-time, and dynamic training protocols covering multiple rehabilitation stages.

The rest of the article is presented as follows: Section 2 provides an overview of the decision model components and the selection of model parameters, Section 3 presents the RBR-based cycle plan inference model construction, Section 4 presents the CBR-based training parameter inference model construction, Section Section 5 presents validation and result, Section 6 and Section 7 discussions and conclusion respectively.

## 2 Decision modeling base on RBR and CBR

This section is presented in two pieces: the first being the determination of the training program generation route and the second presenting the determination of the model parameters.

### 2.1 Training program generation route

**FIGURE 1:**
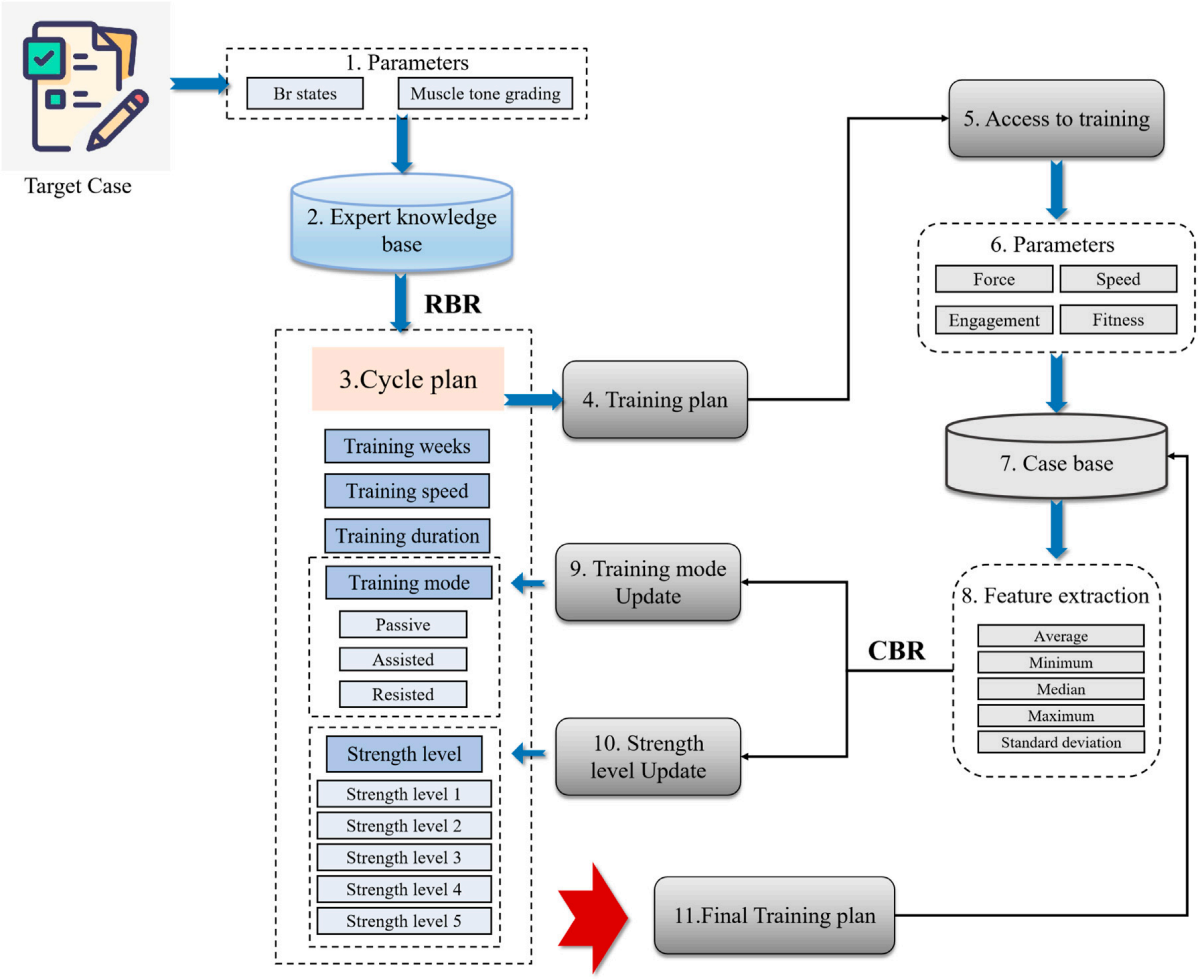
Training program generation route based on CBR-RBR fusion. (1) Determined input parameters through expert research. (2) Constructed the expert knowledge base. (3) Built an RBR cycle plan inference model. (4) Outputted a preliminary cycle training plan. (5) The patient starts training and parameters is generated. (6) Retrieved similar cases from the case base based on the patient’s training data. (7) Performed feature extraction on the retrieved similar cases. (8) Perform case-based reasoning based on extracted features and solutions for similar cases. (9) Strength level update. (10) Training mode update. (11) Outputted the final solution and entered it into the case base reasoning.

This section introduces the specific process of combining CBR-RBR fusion inference. Figure 1 illustrates the CBR-RBR fusion technology route. As shown in Figure 1, the input of the system the main modeling parameters of the target case, and the output is the training plan. First, the input parameters are reasoned through the RBR model to generate a preliminary cycle plan. Subsequently, the patient starts training and the parameters of the patient’s relevant training state are input into the CBR model for program update. Finally, output the final training plan and add it to the case library.

### 2.2 Model parameterization

In order to determine the input parameters of the DSS, the factors related to the patient’s training effect were analyzed in the form of questionnaire, and the most compatible parameters were selected through expert discussion. Ensuring questionnaire quality involved designing it based on extensive literature review and expert recommendations. Ten experts, comprising professional rehabilitation therapists experienced in desktop-based upper limb rehabilitation robot usage, were sourced from 3 rehabilitation hospitals and 7 general hospitals featuring rehabilitation departments in Shanghai. This study distributed a total of 10 questionnaires, achieving a 100% response rate. The experts’ composition is detailed in Table 1.

**TABLE 1:** Composition of experts group.

Category		Experts	Composition ratio (n = 10) (%)
Years in the field	≤ 5 years	4	40
	>5 years	6	60
Hospital level	II	2	20
	III	8	80
Using frequency (per week)	≤ 5 times	3	30
	>5 times	7	70

After examining the collected questionnaires, results indicated unanimous selection by experts of the Brunnstrom stage and muscle tone for determining the robot’s training mode. As for determining the robot’s strength level, nine experts recognized engagement and the fitness, while seven experts supported the speed and force of the affected limb’s movement (several experts endorsed all four parameters simultaneously). Consequently, the Brunnstrom stage and muscle tone will serve as input parameters for determining the robot’s training mode in this study. Meanwhile, engagement, fitness, affected limb movement speed, and force during training will be pivotal input parameters for determining the robot’s strength level. Since training speed and training duration often assume fixed values, our DSS integrated values commonly practiced by therapists in clinical settings.

Engagement reflects the degree of subject’s engagement in the training process. In order to quantify the degree of engagement in the collaborative process, this paper define engagement as the percentage of the actual work done by the subject to complete one training task arm *versus* its required work:

$$Engagement=\frac{W_{\text{user }}}{W_{\text{arm }}}\times 100\%=\frac{W_{\text{total }}-W_{\text{motor }}}{W_{\text{total }}-W_{\text{robot }}}\times 100\%$$ (1)

Where *W*_user_ is the work by subject’s arm during the actual training process, *W*_arm_ is the work required to complete the training task subject’s arm alone, *W*_total_ is the sum of the work by the robot and the subject’s arm without exerting force when completing a training task, *W*_motor_ is the work by robot during the actual training process, *W*_robot_ is the work by the robot running unloaded.

The fitness of the training trajectory is the degree of consistency between the actual movement trajectory and the preset trajectory, and the higher fitness represents the better subject’s upper limb motor coordination and control ability. The formula for calculating fitness is as follows:

$$R^{2}=1-\frac{\sum_{i=1}^{n}{\left(y_{i}-{\hat{y}}_{i}\right)}^{2}}{\sum_{i=1}^{n}{\left(y_{i}-\bar{y}\right)}^{2}}$$ (2)

$$Fitness=R^{2}\times 100\%$$ (3)

Where *y*_*i*_ represents the actual values, ${\hat{y}}_{i}$ represents the preset values, $\bar{y}$ is the mean of the observed values, *n* is the number of observations.

## 3 Cycle plan inference model construction based on RBR

**FIGURE 2:**
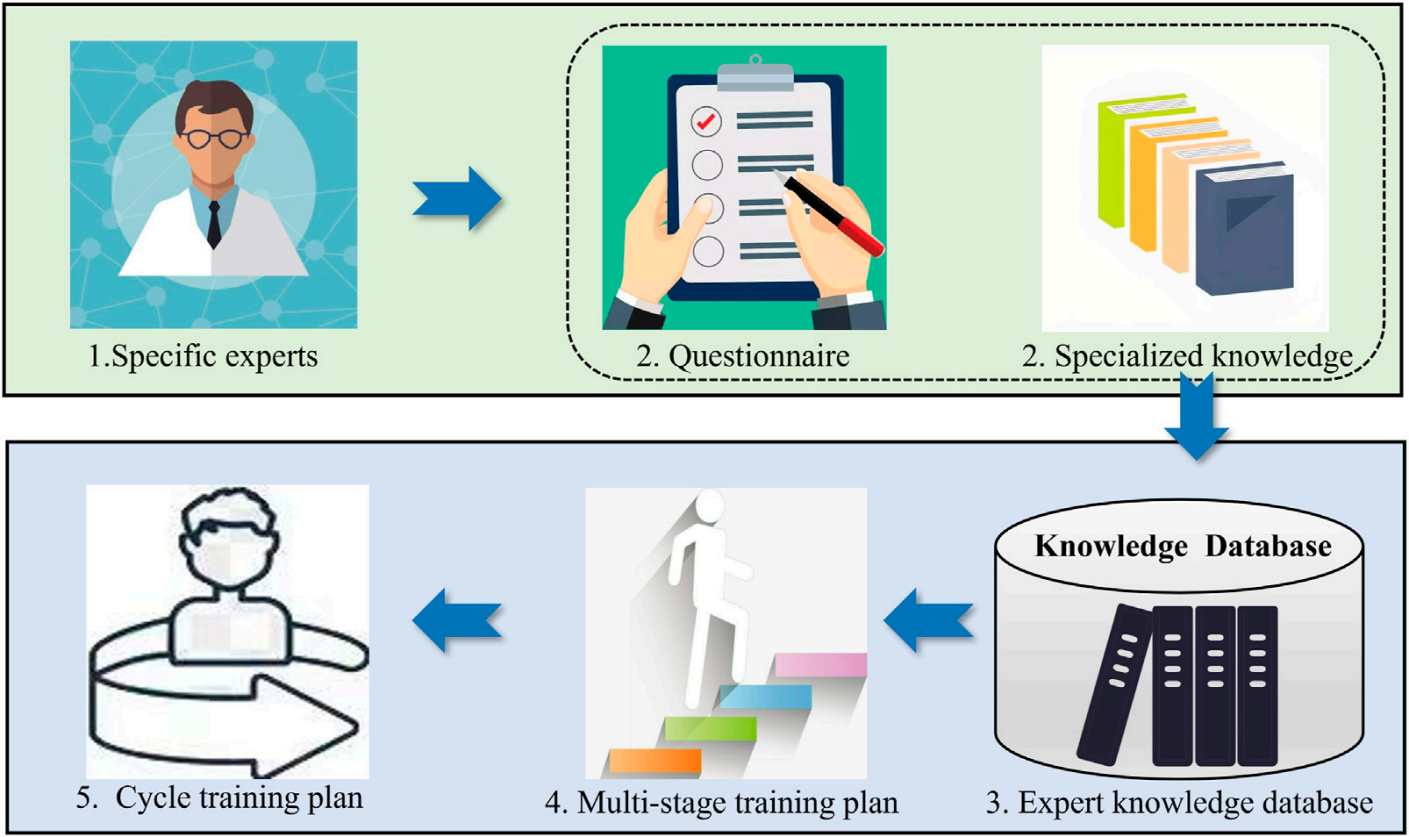
Construction process for cycle plan inference model.

Firstly, suitable experts are selected to generate the previous questionnaire and organize the entries into entries with professional knowledge, thus constructing a rule base for cycle plan reasoning (expert knowledge base). Then, based on the expert knowledge base, the reasoning relationship between the patient’s condition and the training plan is analyzed, so as to establish a cycle plan reasoning model based on RBR. This model will outline the overall approximate training plan for the patient over a 21-day period, with each day’s training plan further divided into four phases, and then establish a DSS for rehabilitation training that combines the long and short periods. The flowchart illustrates as Figure 2: 1. Selection of experts for research; 2. Acquisition of questionnaires and relevant professional knowledge; 3. Organization into an expert knowledge database; 4. Attainment of a multi-stage training plan; 5. Formation of a cycle training plan from the multi-stage plan.

### 3.1 Expert knowledge base

In order to support long-term tracking of patient training decisions, this study employed a previous questionnaire to establish an expert knowledge base, which served as an input database for the cycle plan inference model. The approach aimed to incorporate various stages of the patient’s rehabilitation process into the training plan.

As the rehabilitation robot primarily caters to patients with Brunnstrom stages ranging from II to V, this study aimed to collect and analyze case data for them. The cycle plan of this study encapsulates: training mode, training strength level, training weeks, training speed, and training duration. The analysis of the previous questionnaire indicates that Brunnstrom stages and muscle tone can serve as input parameters for determining the training mode. The joint range of motion, muscle tone status, training strength level (as the scale progresses from F1 to F5, the strength level increases), as well as muscle tone grading and training mode that may correspond to patients in different Brunnstrom stages were obtained based on relevant literature research and expert interview discussions, as shown in Table 2.

**TABLE 2:** Patient assessment results Corresponding to training modes.

Br stage	Joint range of motion	Muscle tone status	Muscle tone grading	Training Mode (Training strength level)
II	Mild restriction,<50%	Mild Spasm	0	Passive (F2)
			1/1+/2	Passive (F3)
III	Restricted range of motion	Spasm Exacerbation	3/4	Assist (F5)
			1/1+/2	Assist (F2)
IV	Partial separation,>50%	Spasm Weakening	1/1+/2	Resistance (F2)
V	Separation	Spasm Weakening	1/1+/2	Resistance (F3)

Studies have shown that 1–2 weeks after the onset of stroke represent the acute phase, equivalent to Brunnstrom stages I and II; 3–4 weeks post-stroke indicate the early recovery stage, equivalent to Brunnstrom stages II and III; 4–12 weeks post-stroke signify the mid-recovery stage, equivalent to Brunnstrom stages III and IV; and 4–6 months post-stroke denote the late recovery stage, equivalent to Brunnstrom stages V and VI for patients (Li et al., 2023). Based on this medical knowledge the number of training weeks under different Brunnstrom stage of the patients was prescribed. Table 3 shows the training weeks rule.

**TABLE 3:** Training weeks rule.

Brunnstrom stage	Muscle tone grading	Training weeks
II	0	1
II	1/1+/2	1
III	3/4	2
III	1/1+/2	2
IV	1/1+/2	5
V	1/1+/2	8

Questionnaire shows that experts are used to setting fixed values for training speed and training duration, 6 of them are used to using level 1 training speed, and 8 of them are used to setting the training duration to 10 min, so the therapist’s commonly used values in the clinic are also used in the DSS. Analyzing and summarizing the above knowledge can get the expert knowledge base as shown in Table 4.

**TABLE 4:** Expert knowledge base.

Project	Judgment (IF)	Conclude (Then)	
Training week	Brunnstrom stage	PhaseIII	2 weeks
		PhaseIII	4 weeks
		PhaseIV	5 weeks
		PhaseV	8 weeks
Training duration	Patient requirements	Warm-up	2min
		Stage 1	10 min
		Stage 2	10 min
		Relaxation	2 min
Training mode (Training strength level)	Brunnstrom stage (Muscle tone grading)	PhaseII(0)	Passive (F2)
		PhaseII(1/1+/2)	Passive (F3)
		PhaseIII(3/4)	Assist (F5)
		PhaseIII(1/1+/2)	Assist (F2)
		PhaseIV	Resistance (F2)
		PhaseV	Resistance (F2)

### 3.2 Construction of cycle plan inference mode

In Section 3.1, the expert knowledge base for the cycle plan reasoning model was established, and the expert knowledge about the reasoning training mode, training strength, training weeks, training speed, and training duration has been collected. To simulate therapists’ cognitive processes in resolving rehabilitation decision-making dilemmas, the RBR method was selected to construct a cycle plan inference model aimed at formulating a multi-stage training program for patients.

According to literature research and hospital visits, therapists develop a two-stage training program in order to enhance the patient’s motivation and multidimensional capability (Rosenbaum and Hennig, 1995). Hence, the system develops a multi-stage training plan for patients that follows the rule of “Warm-up - Stage 1 - Stage 2 - Relaxation”.

The specific rules of the multi-stage training program are as follows: the initial stage employs a 2-min warm-up period, allowing the patient to transition into the training state; the second and third stages utilize identical training modes and strength, targeting diverse joint mobility and muscles with varied training trajectories, each episodelasting 10 min based on clinical expertise. The fourth stage implements a 2-min relaxation mode to alleviate muscle tension and restore them to their baseline. During warm-up and relaxation, the passive mode’s lowest difficulty level (level 1) was chosen to facilitate without increasing muscle training stress.

Per medical insurance regulations, the number of training days for a patient within a single rehabilitation hospital is limited to 21 days. Upon reaching this number, patients may be transferred to another facility. Hence, the cycle plan inference model of this system devises a training plan for the patient spanning 21 days.

The cycle plan inference rule base, derived from the aforementioned rule summary analysis, is presented in Table 5, where the ‘Rehabilitation stages’ include ‘Brunnstrom stage and Muscle tone grading’. After the patient completes the Brunnstrom stages and muscle tone assessment, this rule base selection aligns with the rehabilitation stage of the training program. It integrates with the training parameter inference model during the training process, adapting the program according to the patient’s training status.

**TABLE 5:** Cycle plan inference rule base.

Rehabilitation stage	Weeks	Warm-up (2min)	Stage 1 (10min)	Stage 2 (10min)	Relaxation (2min)
PhaseII(0)	1	Passive (F1)	Passive (F2)	Passive (F2)	Passive (F1)
PhaseII(1/1+/2)	1	Passive (F1)	Passive (F3)	Passive (F3)	Passive (F1)
PhaseIII(3/4)	2	Passive (F1)	Assist (F5)	Assist (F5)	Passive (F1)
PhaseIII(1/1+/2)	2	Passive (F1)	Assist (F2)	Assist (F2)	Passive (F1)
PhaseIV	5	Passive (F1)	Resistance (F2)	Resistance (F2)	Passive (F1)
PhaseV	8	Passive (F1)	Resistance (F3)	Resistance (F3)	Passive (F1)

## 4 Training parametric inference model construction based on CBR

**FIGURE 3:**
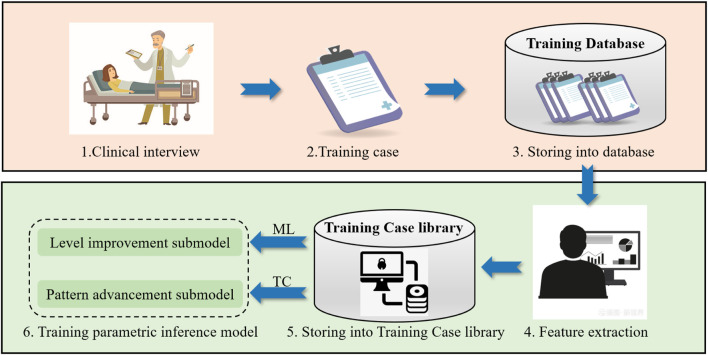
Construction process for training parametric inference models.

As shown in Figure 3, construction process for training parametric inference models, which consists of the following six main steps: 1. Conduct clinical interviews to research patient conditions; 2. Generate training case bases on patient conditions; 3. Develop the training case base; 4. Feature extraction; 5. Develop the training case library; 6. Build the training parametric inference model with two submodels: level improvement submodel and pattern advancement submodel, which are built by different methods, where ML is machine learning, TC is threshold control.

### 4.1 Training case feature library

Based on the analysis in Section 2.2, the clinical case data including engagement, fitness, movement speed of the affected limb, and force of the affected limb during training can be used as inputs to the training parameter inference model. We established the input database for the training parameter inference model based on the clinical case data collected in the hospital, enabling the system to dynamically adjust training parameters according to the patient’s rehabilitation progress.

Training cases involving patients using the desktop-based upper limb rehabilitation robot were gathered from hospitals that integrated it into their routine training regimens. The patient’s condition was examined in the preliminary stage, and a case collection form was devised to gather fundamental patient information. The case collection form is shown in Table 6.

**TABLE 6:** Case collection form.

Attribute	Value	Example
Name	-	Zhang san
Age	-	50
Gender	Male/Female	male
Disease	Ischemic stroke/Hemorrhagic stroke	Ischemic stroke
Affected side	Left/Right	Left side
Brunnstrom stages	Phase II/III/IV/V	Phase III
Muscle strength	Level 0/1/2/3/4/5	Level 1
Muscle tone	Level 0/1/1+/2/3/4	Level 1
Range of motion	Small/Medium/Large	Medium
Date of joining (machine use)	-	2022.9.1
Total usage days	-	210 days
Passive mode usage duration	-	870min
Assistance mode usage duration	-	3100min
Resistance mode usage duration	-	0min

This study collected basic information and training case data from 54 stroke patients, comprising 24 females and 30 males, with a mean age of 60.2 ± 29.2 years. All cases met specific criteria: 1) Upper limb motor dysfunction due to stroke; 2) Brunnstrom stages assessed between phases II to V; 3) Training within a 2-year period; 4) Absence of visual or auditory impairment; 5) No comprehension deficits. Table 7 presents information on these 54 cases. It is worth noting that the same person may have undergone training using multiple modes, and where ‘Left’ is the affected left side, and ‘Right’ is the affected right side.

**TABLE 7:** Case information situation.

Mode	Patients	Male	Female	Left	Right
Passive	26	13	13	9	17
Assisted	38	20	18	13	25
Resisted	21	13	8	7	14

The training case data comprised four parameters: the patient’s engagement, fitness, movement speed of the affected limb, and force of the affected limb in passive, assistance, and resistance modes. MATLAB was used to extract features such as mean, median, maximum, minimum, and standard deviation from the training case data in passive, assisted, and resistance modes. This process resulted in a total of 20 features.

For each training session, the raw data amounts to 120,00 × 4, resulting in a total of 14,782 training sessions. Post feature processing, 2,676 sets of sample data were derived, including 1,020 sets for passive mode, 1,162 sets for assisted mode, and 494 sets for resistance mode. Table 8 displays the creation of the case feature library.

**TABLE 8:** Case feature library.

Data category	Project content	Example
Average value	Average force	1.033
	Average velocity	0.073
	Average fit rate	77.631
	Average engagement	37.316
Maximum value	Maximum force	4.695
	Maximum velocity	0.141
	Maximum fit rate	99.684
	Maximum engagement	100.000
Minimum value	Minimum force	0.000
	Minimum velocity	0.003
	Minimum fit rate	0.000
	Minimum engagement	0.000
Standard deviation	Standard deviation force	1.053
	Standard deviation velocity	0.026
	Standard deviation fit rate	15.950
	Standard deviation engagement	22.505
Median	Median force	0.656
	Median velocity	0.074
	Median fit rate	81.485
	Median engagement	32.921

### 4.2 Training parametric inference model construction

We established a case feature library for the training parameter inference model in the previous section. In order to fully explore the potential value information in the clinical case data and improve the decision-making accuracy, a combination of machine learning and CBR was used to construct the training parameter inference model, thus realize the system adaptive adjustment scheme to fit the rehabilitation process.

According to literature research and expert interviews, therapists tend to gradually increase the training difficulty for patients when their abilities increase (Waeber et al., 2015), so as to ensure that patients can progressively enter the next rehabilitation process. We divided the model into two modules: the strength level improvement submodel and the pattern advancement submodel. The strength level improvement submodel learns the case feature library through machine learning algorithms, analyzes whether the patient’s current training status is able to enter the next rehabilitation strength level stage, and controls the progress of the patient’s training strength level promotion. The pattern advancement submodel assesses the specific parameters of the patient’s ongoing training using a threshold control method, regulating the patient’s progression into the subsequent training mode once the parameter reaches a specified threshold.

#### 4.2.1 Strength level improvement submodel

During the actual training process, the expert will decide whether to upgrade the level for the patient based on the patient’s completion of the current level of training, which influenced by subjective factors. To quantitatively analyze the potential logical relationship between training difficulty and the patient’s training status, we apply machine learning algorithms as a research method for level improvement submodel. Diverse machine learning models are established for various training modes (passive mode, assisted mode and resisted mode), and comparative analyses the values of recall rate, accuracy rate, F1 score, and AUC. (This part is detailed in Section 5.1). The final optimal construction process involves initially constructing the machine learning model using Scikit-learn, followed by data normalization, feature selection using Chi-square filtering, and finally integrating with the random forest algorithm to build the strength level improvement submodel.

#### 4.2.2 Pattern advancement submodel

The threshold ranges of the training parameters vary across modes. In passive mode, the affected limb mainly follows the machine movement, so the affected limb has less force and higher fitness; in assisted and resistance modes, the affected limb has more force and higher engagement, but inadequate patient activity accuracy might reduce the fitness level. Therefore, it needs to be modeled separately for different training modes. As machine learning methods are inapplicable for transitioning to the next mode during patient training, the threshold control method was adopted. This method entailed establishing the threshold values of the training parameters by analyzing reference values associated with the patient’s exertion state during training, aiming to facilitate the patient’s progression to the next training mode.

**FIGURE 4:**
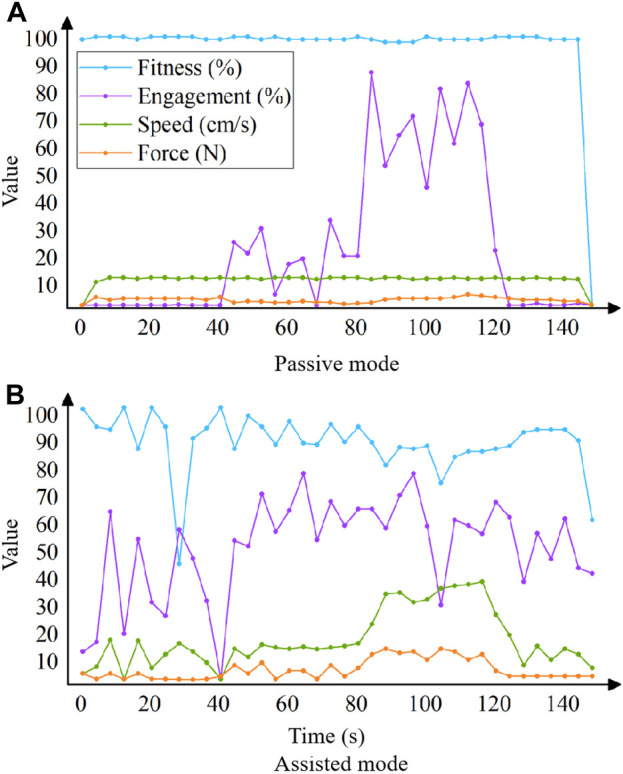
Relationship between exertion state and each training parameter in the passive mode **(A)** and assisted mode **(B)**.

Experimentally verifying the training parameters that are more correlated with the state of exertion, gradually increasing the amount of force exerted by the upper limb on the handle in the passive and assisted modes, and plotting the line graphs to obtain the changes in the four training parameters of engagement, fitness, movement speed of the affected limb, and force of the affected limb. As shown in Figure 4, where ‘Force’ is force of the affected limb, ‘Speed’ is movement speed of the affected limb.

According to Figure 4, it can be obtained that a significant positive correlation trend between robot’s strength level and engagement, while force of the affected limb shows a smaller positive correlation trend, with no significant correlation trend in the fitness and engagement; in the assistance mode, there is a positive correlation between the force and speed, while the force shows a relatively small positive correlation, with no significant correlation trend in the fitness and engagement. As a result, the selection of engagement as input parameter in the passive mode, and progression to the assisted mode when the patient’s average engagement in training was greater than or equal to 70%; choose the movement speed as the input parameter in the assisted mode, by analyzing the speed curve in the figure, when the time is 50–80 s, the speed is stable at 15 cm/s, when the time is 80–120 s, the speed rises sharply and stabilizes at about 35 cm/s, so take the intermediate value of 25 cm/s as the critical value, i.e., when the patient’s average movement speed in training is greater than or equal to 25 cm/s, the progression to resistance mode.

## 5 Validation and results

We validate our DSS in two parts: strength level improvement submodel validation and rehabilitation program decision-making experiment. To validate the strength level improvement submodel, we choose recall, accuracy, F1 score and AUC for comparative evaluation. As shown in Section5.1. In order to test the decision-making effect of the system in the clinic, we selected subjects who met the requirements for clinical testing, recorded the intelligent decision-making results, and then compared the intelligent decision-making results with the expert decision-making results for a comparative study. As shown in Section5.2.

### 5.1 Strength level improvement submodel validation

The establishment of the training parameter inference model has been concluded in Section 4.2. This model delineates two modules: the strength level improvement submodel and the pattern advancement submodel. The latter heavily relies on threshold control, previously analyzed in Section 4.2.2. Hence, Section 4.1 is dedicated to validating the strength level improvement submodel.

**FIGURE 5:**
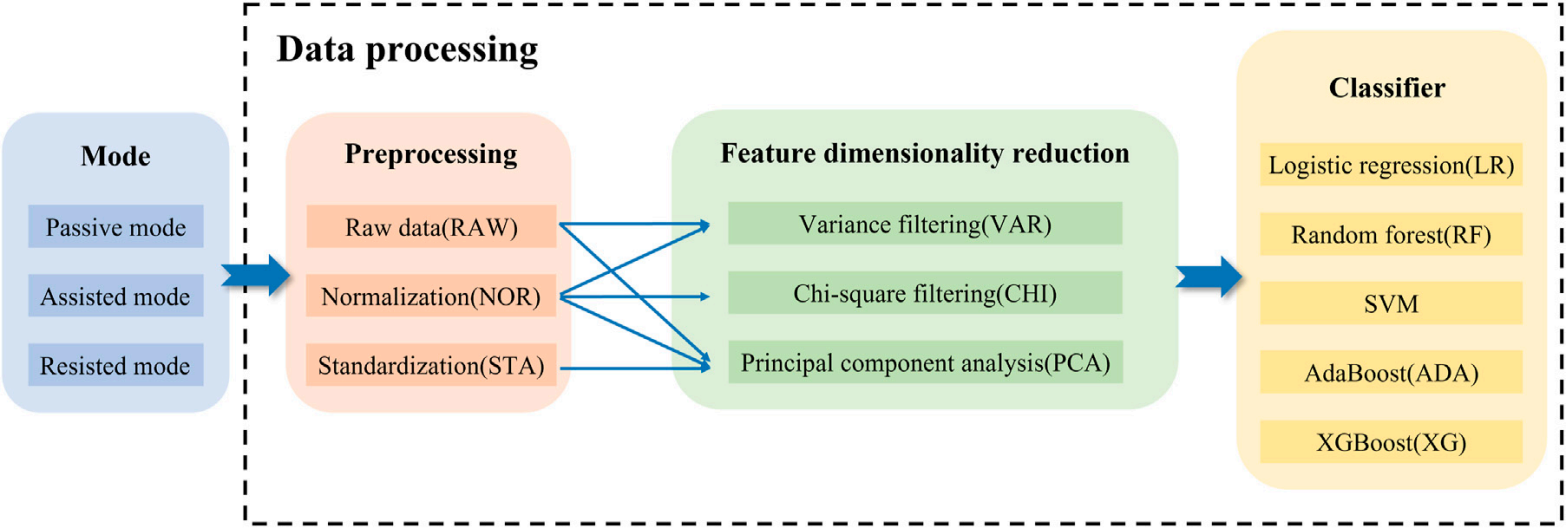
Algorithm fusion comparison experimental design.

In this study is mainly the strength level improvement submodel constructed based on the machine learning method of data normalization-chi-square filtering-random forest, and in order to verify the superiority of this submodel, this part designs the algorithm fusion comparison experiment. Upon evaluating the data type and volume within the case base constructed in Section 4.1, logistic regression (LR), random forest (RF), support vector machine (SVM), AdaBoost (ADA), and XGBoost (XG) were selected. These machine learning algorithms combined with various data preprocessing and Feature dimensionality reduction methods to comparatively analyze their effects. The specific process is shown in Figure 5: After obtaining the data from the training case feature library, data preprocessing is performed, followed by feature dimensionality reduction, and finally different machine learning algorithms are performed to obtain different algorithmic fusion schemes.

Tuning hyperparameters in algorithms to optimize model parameters by considering generalization error and model complexity is crucial in machine learning. Generalization error, a key metric, gauges model accuracy; higher generalization error indicates reduced model effectiveness. There exists a strong correlation between generalization error and model complexity. When the model is overly simplistic, generalization error increases due to underfitting, and when excessively complex, it rises due to overfitting. Optimal performance occurs when the model complexity is appropriately balanced. Thus, this study refines hyperparameter values by analyzing the interplay between generalization error and model complexity. Individual algorithms undergo hyperparameter tuning, and the specific values are detailed in Table 9.

**TABLE 9:** Hyperparameter values.

Filtering	Hyper-parameters	Values
Logistic regression	Penalty Parameter	l2
	Solver for Optimization	liblinear
	Regularization Parameter	0.1
	Max Iterations	1,000.0
Random forest	Number of Trees	54.0
	Random Number Generator Seed	70.0
	Maximum Depth of Trees	16.0
SVM	Kernel Type	rbf
	Kernel Parameters	auto
	Cache Size	5000.0
AdaBoost	Maximum Depth of Trees	16.0
	Number of estimators	1,000.0
	Learning Rate	3.0
XGBoost	Number of Estimators	340.0
	Column Subsampling Ratio for Trees	0.6
	Learning Rate	0.3
	Maximum Depth of Trees	3.0
	Subsample	0.7

Following the selection of optimal hyperparameter values for each algorithm, a fusion of various data preprocessing methods, feature dimensionality reduction techniques, and hyperparameter-optimized algorithms is employed to determine the algorithm combination yielding the most effective classification. Raw data typically contains varying data specifications, and numerical differences between features may compromise classification accuracy. The process of transforming diverse data specifications into a standardized or specific distribution in machine learning is known as “dimensionless scaling”. Common dimensionless methods encompass data normalization and standardization, ensuring uniformity across all data specifications (Nan et al., 2022).

The process of data normalization is to center the minimum value and then scaling it based on the extreme deviation (Pua et al., 2020). The formula for data normalization is as follows, where *x* is the original data, min(*x*)is the minimum value of the original data, max(*x*) is the maximum value of the original data, and *x*′ is the normalized data:

$$x^{\prime }=\frac{x-\min\left(x\right)}{\max\left(x\right)-\min\left(x\right)}$$ (4)

The process of data standardization is to center the data based on the mean, and then scale it based on the standard deviation (Ji et al., 2021). The formula for data standardization is as follows, where*x*is the raw data, *μ* is the mean, *σ* is the standard deviation, and *x*′ is the normalized data:

$$x^{\prime }=\frac{x-\mu }{\sigma }$$ (5)

Feature selection is an important task in machine learning, where irrelevant and redundant features are eliminated by feature selection to improve the learning performance (Wang et al., 2020). In this study, 20 features were selected as inputs to the model in the preliminary stage, and further feature selection is needed for them. The commonly used feature selection methods are filter method, wrapper method, embedded method and dimensionality reduction. Filter method and dimensionality reduction are selected for in-depth algorithmic comparisons based on the data features, so that the optimal combination of features can be selected.

Filtering methods can be further categorized into variance filtering and relevant filtering. Variance filtering (VAR) is a method of filtering by the variance of the features themselves (Zhou et al., 2020). Its variance is calculated as follows, where *X* is the feature matrix and *p* is the probability of one of the classes in that feature:

$$Var\left[X\right]=p\left(1-p\right)$$ (6)

Relevant filtering can filter out features that are more relevant and meaningful to labels (KIYAK et al., 2021). This article selects chi square filtering as one of the alternative feature selection methods. The calculation formula is as follows, where *O*_*i*_ is the observation frequency, *E*_*i*_ is the expected frequency:

$$x^{2}=\overset{k}{\underset{i=1}{\sum }}\frac{{\left(O_{i}-E_{i}\right)}^{2}}{E_{i}}$$ (7)

The essence of feature selection in dimensionality reduction method is matrix decomposition (Taherkhani et al., 2020). In this paper, principal component analysis (PCA) is chosen as one of the feature selection methods. The sample variance formula is as follows, where n is the number of samples,*x*_*i*_ is the sample value, $\bar{x}$ is sample mean:

$$Var=\frac{1}{n-1}\overset{n}{\underset{i=1}{\sum }}{\left(x_{i}-\bar{x}\right)}^{2}$$ (8)

**FIGURE 6:**
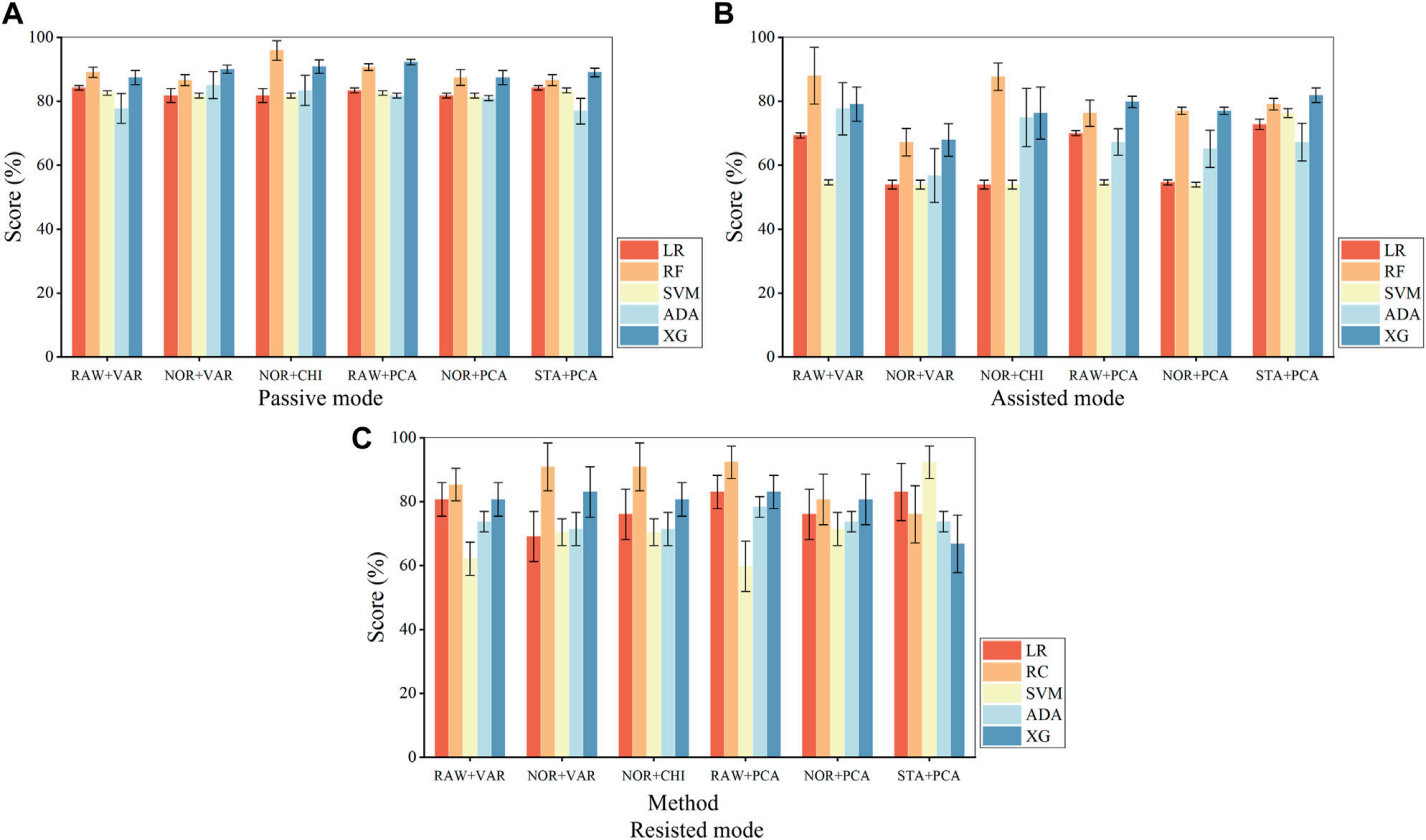
Fusion algorithm accuracy in the passive mode **(A)**, assisted mode **(B)**, and resisted mode **(C)**. where ‘RAW + VAR’ is ‘raw data + variance filtering’, ‘NOR + VAR’ is ‘normalization + variance filtering’, ‘NOR + CHI’ is ‘normalized + chi-square filtering’, ‘RAW + PCA’ is ‘raw data + principal component analysis’, ‘NOR + PCA’ is ‘normalization + principal component analysis’, ‘STA + PCA’ is ‘standardization + principal component analysis’, ‘LR’ is ‘logistic regression’, ‘RF’ is ‘random forest’, ‘ADA’ is ‘AdaBoost’, ‘XG’ is ‘XGBoost’.

Evaluate the algorithm’s classification performance based on classification accuracy and AUC value. In classification, the accuracy rate (the proportion of results predicted correctly by the model), characterizes the overall correctness of the classifier (Zhang et al., 2020). The AUC value (area under the ROC curve) characterizes the performance of the classification model, while the ROC curve shows the threshold effect of the model under all classification categories (Agrawal et al., 2022). The combined step of data preprocessing and feature selection methods (Remeseiro and Bolon-Canedo, 2019), which can also be referred to as feature processing methods. The algorithms with outstanding classification performance were first filtered on each feature processing method and then further filtered using AUC values. Apply a feature processing method to each machine learning algorithm, according to different training modes for model training, respectively, and plot the bar graph to get the classification accuracy as shown in Figure 6.

Based on the bar charts, the top three feature processing and algorithm fusion methods in terms of accuracy in each mode are found, as shown in. It can be obtained that both Random Forest and XGBoost achieve the training effect of ranking the top three in three modes, and their average accuracy in various data processing methods remains above 75%, and then further screening is carried out in these two algorithms. Table 10 shows top 3 fusion algorithm accuracy comparisons for different training modes.

**TABLE 10:** Comparison of fusion algorithm accuracy under different training modes.

Mode	Algorithm	Accuracy (%)
Passive	XGBoost	89.5
	Random Forest	89.4
	Logistic regression	82.9
Assisted	Random Forest	79.2
	XGBoost	77.0
	AdaBoost	68.2
Resisted	Random Forest	86.1
	XGBoost	79.2
	Logistic regression	78.0

**FIGURE 7:**
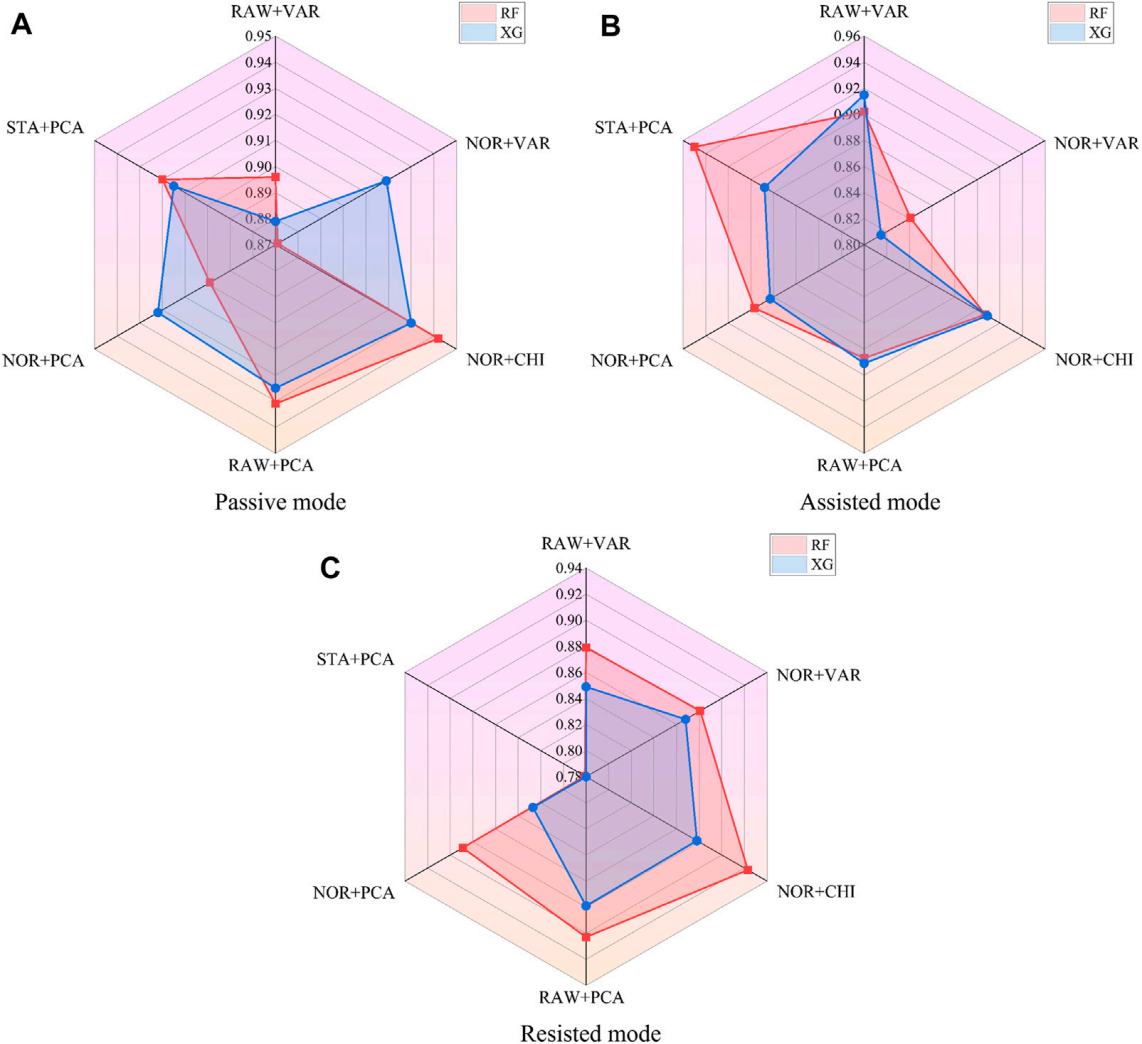
AUC values of fusion algorithms in the passive mode **(A)**, assisted mode **(B)**, and resisted mode **(C)**.

The different feature processing methods are applied to Random Forest and XGBoost, respectively, for the computation of AUC values, as shown in Figure 7. Where the radar scale of the radargram corresponds to the AUC value, the radargram has a total of six radar axes, which represent six different algorithm fusion methods.

Based on the radargrams, the top three feature processing and algorithm fusion methods for AUC values in each mode are found, as shown in Table 11. Where ‘RF’ is the random forest classifier and ‘XG’ is the XGBoost classifier. It can be obtained that RF [NOR + CHI] (Data normalization and chi-square filtering as feature processing, combined with random forest algorithm) achieves the training effect of ranking the top three in all three modes, and all of their AUC value remain above 0.9.

**TABLE 11:** Comparison of AUC values of fusion algorithms under different training modes.

Mode	Algorithm	AUC
Passive	**RF[NOR + CHI]**	**0.942**
	RF [RAW + PCA]	0.931
	XG [NOR + CHI]	0.930
Assisted	XG [RAW + VAR]	0.915
	XG [NOR + CHI]	0.909
	**RF[NOR + CHI]**	**0.907**
Resisted	**RF[NOR + CHI]**	**0.923**
	RF [RAW + PCA]	0.903
	RF [NOR + PCA]	0.889

Bold implies that the RF [NOR + CHI], AUC values in different modes are in the top three.

According to the above analysis, the data preprocessing method adopts normalization, the feature selection method adopts chi-square filtering, and finally combines with the random forest algorithm to establish the training parameter inference model. After chi-square filtering, feature selection is completed. When the hyper-parameter k is 19, the number of sub classifiers in the random forest reaches 54, and the maximum depth of the model reaches 16, the model can achieve the optimal classification effect. The metrics of each model are shown in Table 12, and the average accuracy of the system is 91.5%, the average AUC value is 0.924, the average recall is 88.7%, and the average F1 score is 90.1%.

**TABLE 12:** Indicators in each mode.

	Accuracy (%)	AUC	Recall (%)	F1 score (%)
Passive	95.9	0.942	90.9	94.1
Assisted	87.7	0.907	86.7	85.4
Resisted	90.9	0.923	88.5	90.8
**Average**	**91.5**	**0.924**	**88.7**	**90.1**

The mean value of system performance is deepened with bold.

### 5.2 Rehabilitation program decision-making experiment

In order to test the effectiveness of the system in clinical decision making, 15 stroke subjects were convened to train with the desktop-based upper limb rehabilitation robot for 30 sessions, including 7 females and 8 males, with a mean age of 57.5 ± 25.5 years. The desktop-based upper limb rehabilitation robot in our study called Armguider produced by Shanghai ZhuoDao Medical Technology Co. All subjects met the following criteria: 1) Upper limb motor dysfunction caused by stroke; 2) The Brunnstrom stages evaluation results are from II to IV; 3) No visual or auditory impairment; 4) No comprehension barriers, able to understand experimental requirements. The training information of 15 subjects is shown in Table 13, where types 1 to 3 represent separately passive mode, assistance mode, and resistance mode.

**TABLE 13:** Subject information.

Mode	Subjects	Male	Female	Affected limb site
Passive	4	1	3	Upper limb
Assisted	7	4	3	Upper limb
Resisted	4	3	1	Upper limb

**FIGURE 8:**
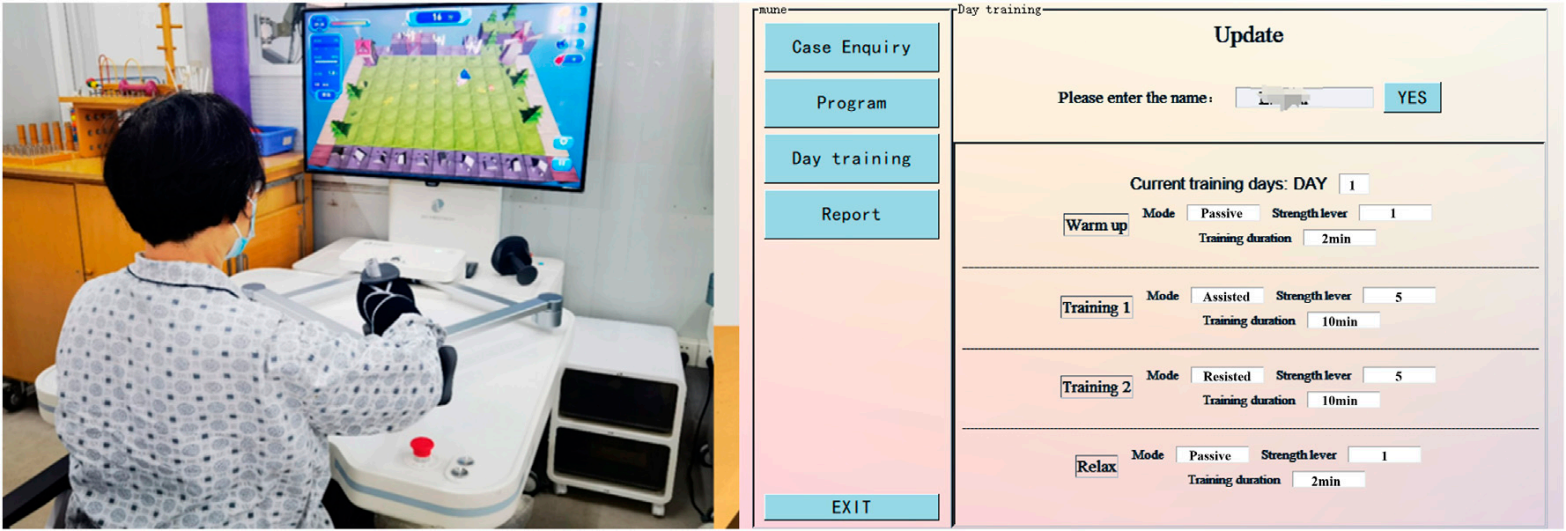
Clinical rehabilitation decision-making experimental scenarios.

Before the experiment began, all subjects were aware of the process and precautions of this experiment and voluntarily signed an informed consent form. The clinical rehabilitation decision-making experimental scenarios is shown in Figure 8.

**FIGURE 9:**
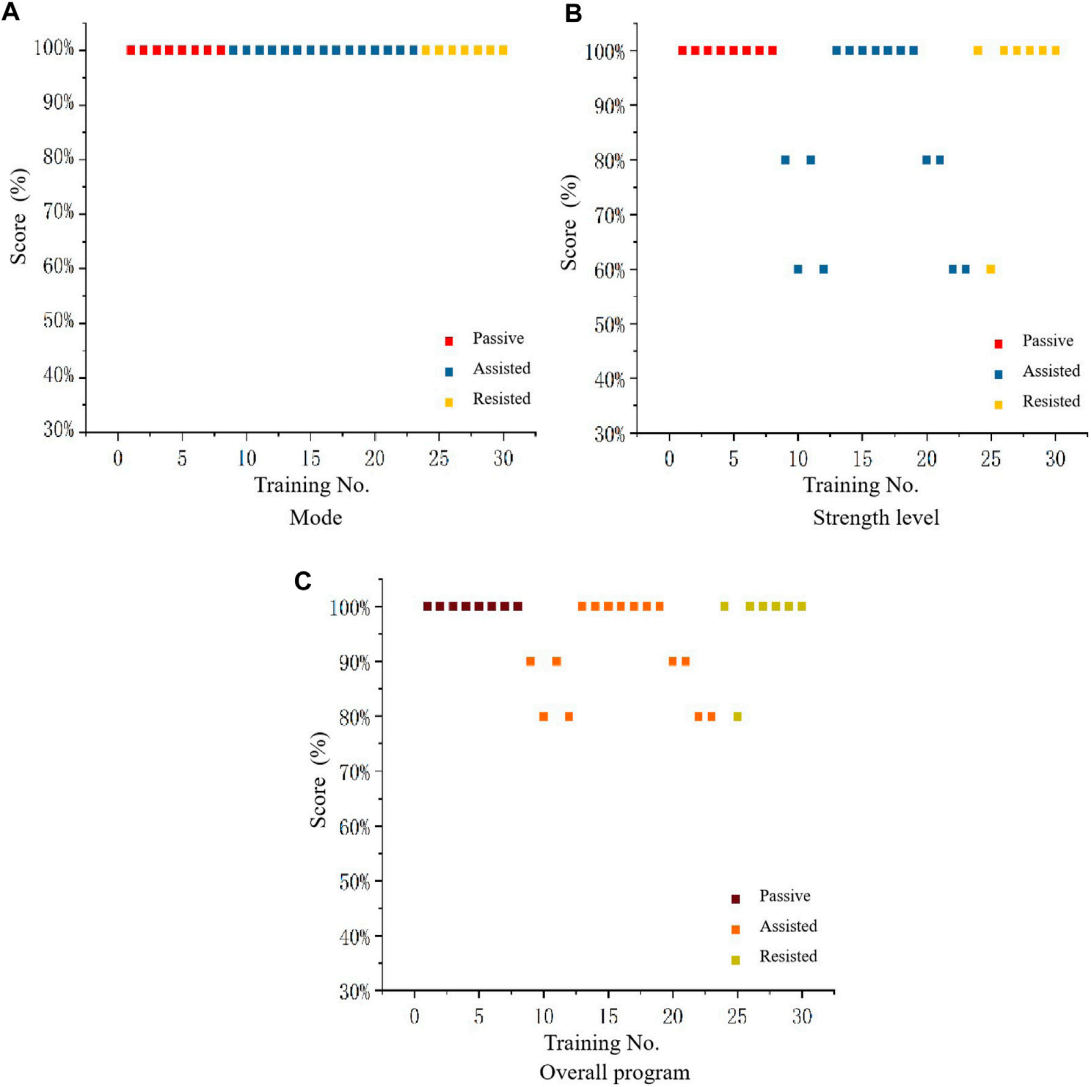
Scatterplot of feasibility evaluation of intelligent decision-making for mode **(A)**, strength level **(B)**, and overall program **(C)**.

In order to verify the feasibility of the system in clinical decision making, experts were asked to evaluate the training programs inferred from the model. The evaluation method was as follows: the experts scored each training program based on the safety and reasonableness of the mode and strength level inferred from each training in actual clinical decision making. As shown in Table 14. Where “Training No. x” is the training serial number (30 training sessions for 15 individuals), E1-E10 are 10 experts. Each expert scores the mode (full marks is 5), strength level (full marks is 5) of reasoning for each training session, and overall program (full marks is 10). The specific scoring details are shown in the following table as an example for a particular patient’s training. The full marks of each training session is 100 out of 100, and the current score is 90. This includes a score of 50 out of 50 for the predicted score of the training mode, and a score of 40 out of 50 for the strength level score. Therefore, the accuracy in terms of score is 90% (Overall program), 100% (Mode), 80% (Strength level). Scatterplot Figure 9 was obtained from the expert rating scale, plotting the mode, robot strength level, and overall evaluation of the program.

**TABLE 14:** The expert rating scale.

Training No.x	DSS	E1	E2	E3	E4	E5	E6	E7	E8	E9	E10	Total marks	Full marks	Score (%)
Mode	Passive	Passive	Passive	Passive	Passive	Passive	Passive	Passive	Passive	Passive	Passive			
Expert rating		5	5	5	5	5	5	5	5	5	5	50	50	100
Strength level	F2	F3	F2	F3	F2	F2	F2	F2	F2	F2	F2			
Expert rating		0	5	0	5	5	5	5	5	5	5	40	50	80
Overall program		5	10	5	10	10	10	10	10	10	10	90	100	90
Expert total rating														

Figure 9A shows that the expert always has a high evaluation of the modes inferred by the DSS, indicating that, it can be seen that the intelligent decision-making model has a high level of feasibility for mode reasoning in rehabilitation training program. By analyzing Figure 9B, it can be concluded that the expert’s evaluation range for the strength level inferred from the system remains between 60% and 100%, with over 80% accounting for the majority, the evaluation of the strength level for passive mode and resistance mode remains almost at a high level, while there are occasional deviations in the assistance mode. Therefore, it can be concluded that the intelligent decision-making model is feasible for strength level reasoning in rehabilitation training programs. By analyzing Figure 9C , it can be concluded that the expert’s evaluation range for the training scheme of system reasoning is maintained between 80% and 100%, with passive mode and resistance mode almost maintaining a high level. Based on the above analysis, it can be concluded that the intelligent rehabilitation DSS has a high feasibility for reasoning the training program.

**FIGURE 10:**
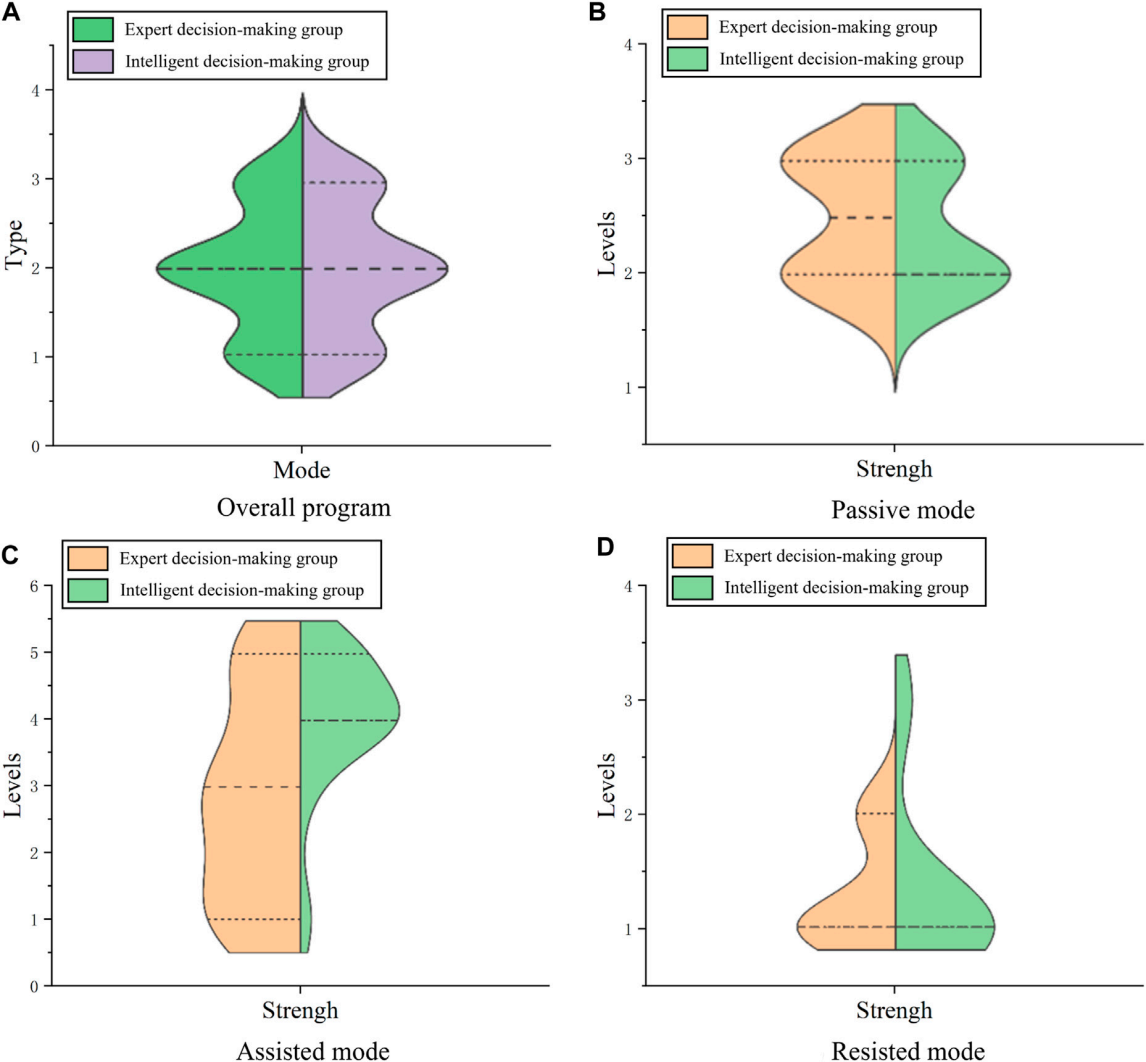
Split-side violin diagrams in the overall program **(A)**, passive mode **(B)**, assisted mode **(C)**, and resisted mode **(D)**.

To verify the decision-making ability of this system in clinical rehabilitation, the training patterns and intensity levels determined by expert and intelligent decision-making in different modes and in the same modes were compared. Divide the data results of the expert’s decision-making and the data results of the DSS into expert decision-making group and intelligent decision-making group. Draw a split-side violin diagrams as shown in Figure 10, where the long dashed line represents the median, the short dashed line above the long dashed line represents the 75% quantile, and the short dashed line below the long dashed line represents the 25% quantile.

Upon analyzing Figure 10A representing the mixed training mode, there’s considerable overlap in the quartiles of training mode between the expert decision-making groups and intelligent decision-making groups. This indicates a high degree of similarity between the expert’s decision and the DSS, which indicates that DSS is excellent at determining training mode. Examining Figure 10B for the passive mode, the median strength level for the manual decision-making group is 2.5, while for the intelligent decision-making group is 2. Both groups predominantly fall within the 2 to 3 range, with 75% quartile overlap, revealing the system’s proficiency in deciding strength levels within the passive mode. Analyzing Figure 10C in the assisted mode, 75% quartiles of training force levels overlap between both groups, with slightly larger medians and distributions observed in the intelligent group compared to the manual group. This indicates that the system’s decisions on force levels in the assisted mode may surpass those of manual decisions. However, given that higher assisted levels imply easier training, the intelligent decision results can be safely applied in clinical patient training. Reviewing Figure 10D for the resistance mode, both groups exhibit a median strength level at 1, with the primary distribution also centered around 1. This demonstrates the system’s adeptness in determining strength grades within the resistance mode.

## 6 Discussions

In this study, we propose an RBR and ML-aided CBR to assist physiotherapists in making rehabilitation training decisions in upper limb rehabilitation robot training scenarios. We combined clinical assessment scales such as Brunnstrom scales and muscle tone with upper limb rehabilitation robot assessment indicators to establish a DSS for rehabilitation robot training based on RBR and ML-aided CBR. This has important clinical value and scientific significance for promoting the intelligent system application of rehabilitation robots.

This study employs quantitative parameters derived from multidimensional robot capability assessments to optimize decision-making in prescription. Compared to current rehabilitation robot evaluations that rely solely on clinical scales and single performance dimensions (Wang et al., 2014; Jiang et al., 2022), this study overcomes issues such as the subjectivity of clinical scales and the poor interpretability of single robot evaluation indices. Thus, it provides a theory and method with application value for intelligent prescription decision-making of rehabilitation robot training.

In this study, we established an upper limb rehabilitation robot decision-making model based on hybrid reasoning of CBR and RBR, and used CBR to update the results of optimized RBR, which led to a significant improvement in the reasoning efficiency (Wen et al., 2014; Saraiva et al., 2016), and changed the traditional situation that relied on the scale only for decision-making in rehabilitation training. In real-life rehabilitation training situations, some patients may propose to perform multiple sets of training on a single day (2 min-10min-10min–2 min as a set), at which point the DSS will quickly adjusts the subsequent training program according to the training status, thus reducing the burden of unplanned situations on the therapist. This study uses real patient data, including personal data, signs, symptoms, and diagnoses. Therefore, there will be a large amount of data, and in the face of similar situations, some scholars have suggested in their research that the idea of machine learning can be integrated (Saraiva et al., 2016), and this study successfully practices the strategy of incorporating machine learning, which allows us to process and utilize the data more deeply and fully.

In this study, in order to select a suitable machine learning method to improve CBR, the performance of algorithms such as SVM, Random Forest, and XGBoost are compared by several metrics such as accuracy, average AUC, recall, and F1. The results show that the Random Forest algorithm performs best in this system. Finally, comparing and analyzing the training plan proposed by the experts and the system, the results show that the experts are recognized the rationality of the system, and it can be expected to reduce the therapist’s workload to a certain extent in the subsequent application.

The limitations of this study are related to the size of the expert knowledge base and the case base. Therefore, we plan to expand the research by recruiting diversified experts in the field or in other fields and establishing expert networks, which will in turn expand the content of the rule base. By processing the data of existing samples and employing methods such as Association Rule Mining (Cheng and Wang, 2017) and Network Analysis (Yu et al., 2020), we will further explore the rule relationships between assessment data and prescription results, aiming to refine and enhance the decision outcomes. With the expansion of the rule base, we intend to try ML to enhance RBR (Rieke et al., 2020), so as to reduce the burden and cost of the process of collecting datasets with annotations. Also in future work, we intend to collect more cases. By increasing the number of cases, time-performance could be affected, but there are effective Case Base Maintenance algorithms that could be used to minimize this issue (Smiti and Elouedi, 2011).

## 7 Conclusion

This research established an expert knowledge base based on the clinical experience of rehabilitation therapists and medical data. By employing the RBR method, a cyclic planning inference model was constructed. Utilizing clinical training case data and CBR, various data processing methods and machine learning algorithms were compared and integrated. The algorithms of chi-square filtering and random forest were selected to build a training parameter inference model. Ultimately, a DSS for upper limb rehabilitation robot training based on rules and cases was developed. The feasibility and effective decision-making capability of the system were verified through practical clinical rehabilitation decisions. This system enhances the efficiency and precision of formulating rehabilitation training plans, integrating medical resources to extract valuable information. It represents a breakthrough in the study of integrating artificial intelligence with dynamic rehabilitation decision-making. Simultaneously, it alleviates challenges related to constrained medical resources and the high workload of rehabilitation therapists to a certain extent, thus exhibiting promising application prospects and substantial research implications.

## Data Availability

The raw data supporting the conclusion of this article will be made available by the authors, without undue reservation.
