# Supplementary material for: A decision support system for upper limb rehabilitation robot based on hybrid reasoning with RBR and CBR
Source: Front Bioeng Biotechnol. 2024 Apr 24;12:1400912. doi: 10.3389/fbioe.2024.1400912 (PMC11076720; doi:10.3389/fbioe.2024.1400912)
Supplement: Supplementary file 1 [file Table1.pdf]

Supplementary Material

1 SUPPLEMENTARY TABLES AND FIGURES

1.1 Tables

Table S1. Questionnaire for experts

Num	Question
1	What are the stages of the common rehabilitation process for stroke patients with hemiplegia? What are the rehabilitation therapies used?
2	What aspect of the patient's feedback do you typically feel as you train them?
3	When a patient is given an unarmed muscle strength test, what percentage of the strength of the affected side of a patient with muscle strength classes 1-5 is on his or her healthy side, respectively?
4	Do you think that the patient's engagement, muscle tone, muscle size, speed of movement of the affected limb, fit, and strength of the affected limb during the current training session can be used as a basis for adjusting the training program (training mode, training effort, training speed, training duration, and training trajectory)?
5	Does the training trajectory set for the patient vary for the different modes of the Armguider?
6	When using the Armguider's passive, assisted, and resistance modes, which training trajectories do you choose for your patients?
7	How long does each Br staging typically take to train into the next staging? What factors influence this?
8	How many days a week does a patient train? How many times a day? What factors determine this?
9	How long do I need to warm up and relax during a training session? Can warm-up and relaxation be replaced by the passive training mode of the rehabilitation robot?

Table S2. Composition of experts group

Category		Experts	Composition ratio (n=10)
Years in the field	<= 5 years	4	40%
	> 5 years	6	60%
Hospital level	II	2	20%
	III	8	80%
Using frequency (per week)	<= 5 times	3	30%
	>5 times	7	70%

Table S3. Patient Assessment Results Corresponding to Training Modes

Br stage	Joint range of motion	Muscle tone status	Muscle tone grading	Training Mode(Training strength level)
II	Mild restriction ,<50%	Mild Spasm	0	Passive (F2)
			1/1+/2	Passive (F3)
III	Restricted range of motion	Spasm Exacerbation	3/4	Assist (F5)
			1/1+/2	Assist (F2)
IV	Partial separation,>50%	Spasm Weakening	1/1+/2	Resistance (F2)
V	Separation	Spasm Weakening	1/1+/2	Resistance (F3)

Table S4. Training weeks rule

Brunnstrom stage	Muscle tone grading	Training weeks
II	0	1
II	1/1+/2	1
III	3/4	2
III	1/1+/2	2
IV	1/1+/2	5
V	1/1+/2	8

Table S5. Case collection form

Attribute	Value	Example
Name	-	Zhang san
Age	-	50
Gender	Male/Female	male
Disease	Ischemic stroke/Hemorrhagic stroke	Ischemic stroke
Affected side	Left/Right	Left side
Brunnstrom stages	Phase II/III/IV/V	Phase III
Muscle strength	Level 0/1/2/3/4/5	Level 1
Muscle tone	Level 0/1/1+/2/3/4	Level 1
Range of motion	Small/Medium/Large	Medium
Date of joining (machine use)	-	2022.9.1
Total usage days	-	210 days
Passive mode usage duration	-	870min
Assistance mode usage duration	-	3100min
Resistance mode usage duration	-	0min

Table S6. Case information situation

Mode	Patients	Male	Female	Left	Right
Passive	26	13	13	9	17
Assisted	38	20	18	13	25
Resisted	21	13	8	7	14

Table S7. Hyperparameter values

Filtering	Hyper-parameters	Values
Logistic regression	Penalty Parameter	l2
	Solver for Optimization	liblinear
	Regularization Parameter	0.1
	Max Iterations	1000.0
Random forest	Number of Trees	54.0
	Random Number Generator Seed	70.0
	Maximum Depth of Trees	16.0
SVM	Kernel Type	rbf
	Kernel Parameters	auto
	Cache Size	5000.0
AdaBoost	Maximum Depth of Trees	16.0
	Number of estimators	1000.0
	Learning Rate	3.0
XGBoost	Number of Estimators	340.0
	Column Subsampling Ratio for Trees	0.6
	Learning Rate	0.3
	Maximum Depth of Trees	3.0
	Subsample	0.7

Table S8. Comparison of fusion algorithm accuracy under different training modes

Mode	Algorithm	Accuracy
Passive	XGBoost	89.5%
	Random Forest	89.4%
	Logistic regression	82.9%
Assisted	Random Forest	79.2%
	XGBoost	77.0%
	AdaBoost	68.2%
Resisted	Random Forest	86.1%
	XGBoost	79.2%
	Logistic regression	78.0%

Table S9. Comparison of AUC values of fusion algorithms under different training modes

Mode	Algorithm	AUC
Passive	**RF[NOR+CHI]**	**0.942**
	RF[RAW+PCA]	0.931
	XG[NOR+CHI]	0.930
Assisted	XG[RAW+VAR]	0.915
	XG[NOR+CHI]	0.909
	**RF[NOR+CHI]**	**0.907**
Resisted	**RF[NOR+CHI]**	**0.923**
	RF[RAW+PCA]	0.903
	RF[NOR+PCA]	0.889

Table S10. Subject information

Mode	Subjects	Male	Female	Affected limb site
Passive	4	1	3	Upper limb
Assisted	7	4	3	Upper limb
Resisted	4	3	1	Upper limb
